# Predicting cervical lymph node metastasis in papillary thyroid carcinoma using capsule disruption length measured by 3D-US

**DOI:** 10.1007/s00330-025-12070-6

**Published:** 2025-10-17

**Authors:** Ruyu Liu, Yuxin Jiang, Xingjian Lai, Ying Wang, Luying Gao, Ruina Zhao, Xuehua Xi, Bo Zhang

**Affiliations:** 1 Department of Ultrasound, China–Japan Friendship Hospital (Institute of Clinical Medical Sciences), Chinese Academy of Medical Sciences & Peking Union Medical College, Beijing, China; 2 Department of Ultrasound, Chinese Academy of Medical Sciences & Peking Union Medical College Hospital, Beijing, China; 3 Department of Ultrasound, China–Japan Friendship Hospital, Beijing, China; 4 National Center for Respiratory Medicine; State Key Laboratory of Respiratory Health and Multimorbidity; National Clinical Research Center for Respiratory Diseases; Institute of Respiratory Medicine, Chinese Academy of Medical Sciences; Center of Respiratory Medicine, China–Japan Friendship Hospital, Beijing, China; 5 Chinese Academy of Medical Sciences & Peking Union Medical College, Beijing, China

**Keywords:** Thyroid Cancer, Papillary thyroid carcinoma, Lymph node metastasis, Ultrasonography, Invasion

## Abstract

**Objectives:**

Papillary thyroid carcinoma (PTC) is a prevalent endocrine malignancy with a propensity for lymph node metastasis (LNM). Extrathyroidal extension (ETE) is a key factor in preoperative LNM prediction. The criteria for ultrasound diagnosis of ETE remain controversial. The aim is to determine if the length of capsule disruption (LCD) on three-dimensional ultrasound (3D-US) can predict cervical LNM in PTC patients.

**Material and methods:**

A prospective cohort of 168 patients from Peking Union Medical College Hospital was examined by 3D-US. The LCD was measured using the omniview mode of 3D-US. Statistical analyses included Chi-square tests, *T*-tests, Mann–Whitney tests, ROC curve analysis, and logistic regression analysis.

**Results:**

Of the 126 patients included, 71 had LNM. Younger age, male gender, larger malignant nodules, LCD, echogenic foci, and thyroid capsule invasion were significantly associated with LNM. LCD ≥ 0.42 cm increases LNM risk by 4.097 (*p* < 0.001). A nomogram was constructed incorporating gender, age, maximum diameter of the largest malignant nodule (MDLM), and LCD to estimate the risk of LNM. The accuracy and AUC of the nomogram were 73.0% and 0.795 (0.718–0.873).

**Conclusions:**

LCD on 3D-US is a significant predictor of cervical LNM in PTC patients. This study’s nomogram, based on easily measurable parameters, can help in the preoperative assessment of LNM risk, potentially guiding surgical management.

**Key Points:**

***Question*** *Can the LCD measured by 3D-US predict cervical LNM in PTC*?

***Findings*** *LCD ≥ 0.42 cm on 3D-US increase LNM risk by 4.097-fold. The nomogram with LCD, gender, age, and nodule size shows good predictive ability (AUC = 0.795)*.

***Clinical relevance*** *LCD is a promising predictor of LNM, an alternative to ultrasound thyroid capsule invasion evaluation. The nomogram enables risk-adapted surgery, reducing unnecessary dissection or missed metastases to improve patient outcomes*.

**Graphical Abstract:**

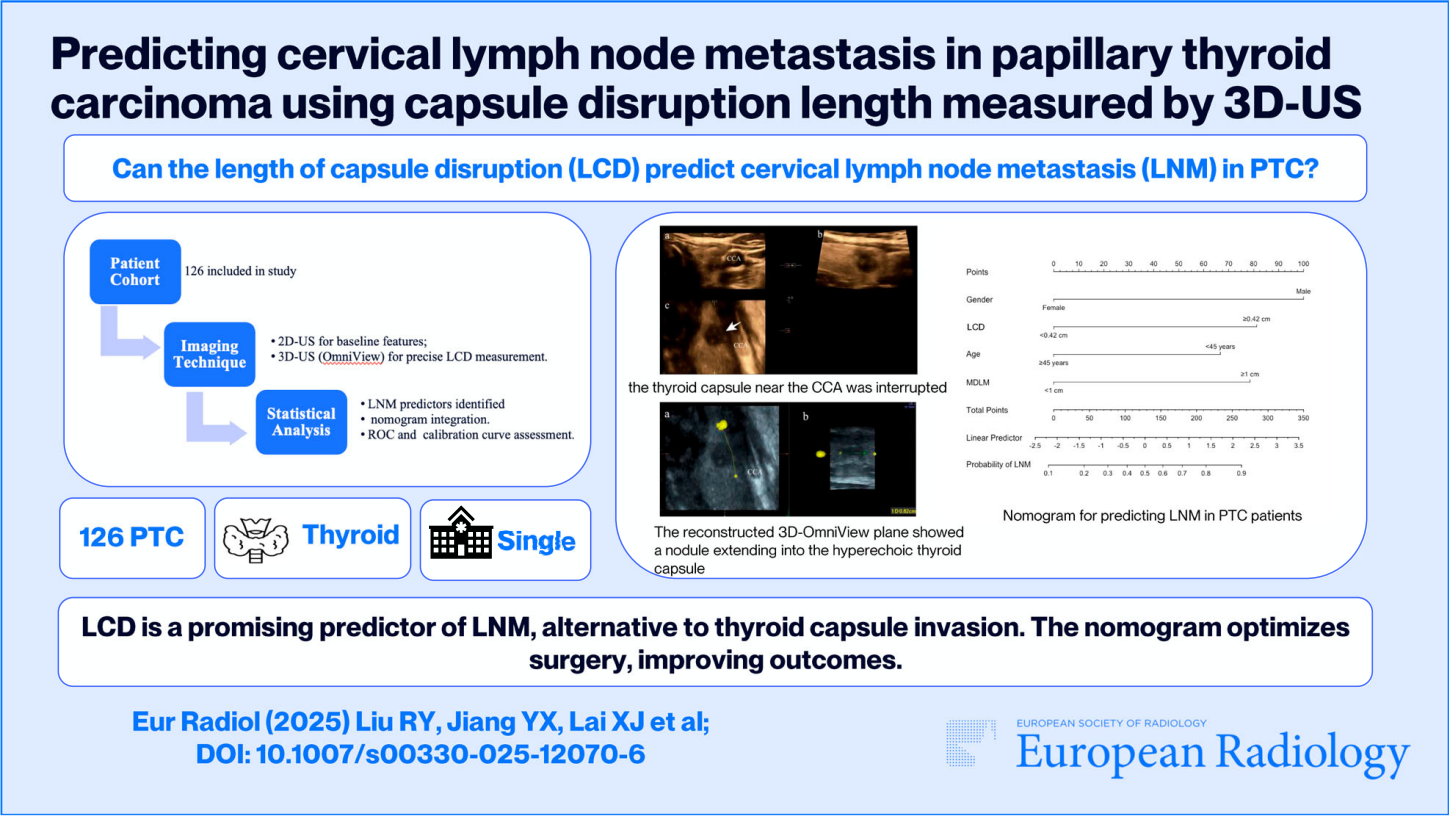

## Introduction

Thyroid cancer (TC) is one of the most common endocrine malignancies worldwide, with an estimated 586,000 new cases reported in 2020 alone [1]. Papillary thyroid carcinoma (PTC) is the predominant histological variant of TC, exhibiting a marked increase in global incidence [2]. Despite its relatively low mortality rate, the rising prevalence poses significant public health challenges, particularly due to the 30–80% possibility for lymph node metastasis (LNM) at initial diagnosis [3, 4], which can adversely affect patient prognosis.

Currently, surgery remains the primary treatment option for PTC, however, controversy persists regarding lymph node management [5]. LNM preoperatively strongly correlates with distant metastasis, high locoregional recurrence, and enhanced death risk [6]. Timely and appropriate surgical intervention, along with thorough dissection of metastatic lymph nodes, can reduce the likelihood of subsequent operations and improve patient prognosis and survival rates [7]. By avoiding unnecessary prophylactic lymph node dissection (LND), the incidence of surgery-related complications, such as hypoparathyroidism, recurrent laryngeal nerve damage, and vocal cord paralysis, can be minimized [8]. Therefore, the early detection and prediction of lymph node involvement is crucial for clinical decision-making and patient prognosis.

Ultrasound (US) has become widely accepted as the first imaging technique in the assessment of cervical LNM of PTC patients preoperatively. CT is recommended as an adjunctive examination for evaluating cervical lymph nodes [9]. CT has a higher sensitivity, while US has a higher specificity for the assessment of cervical LNM. Both the US and CT have limitations in diagnosing cervical LNM, particularly in their ability to accurately identify central LNM [10]. What’s more, most sonographic features of metastatic lymph nodes are atypical, especially in early stages. Even under contrast-enhanced ultrasound, the early LNM are not easy to detect [11].

Various factors have been proposed as predictors of cervical LNM, including extrathyroidal extension (ETE), tumor location, multifocality, age, gender, and nodule size [12]. However, the criteria for ultrasound diagnosis of ETE remain controversial. The diagnostic criteria for ETE on ultrasound vary across studies, ranging from thyroid capsule contact, capsule contact 25–50%, capsule contact > 50%, capsule disruption and protrusion, to invasion of surrounding tissues [13–17]. This lack of a standardized definition can lead to variability in ETE diagnosis, affecting its predictive value for cervical LNM. To address this issue, this study proposes to use the length of capsule disruption (LCD) as a more precise, quantifiable marker for predicting LNM. And in order to precisely measure this length, we employed the omniview mode of three-dimensional ultrasound (3D-US), which offers clearer visualization of the capsule-nodule relationship.

## Materials and methods

### Patients

Between February 2016 and January 2018, a prospective cohort study was conducted at Peking Union Medical College Hospital, enrolling 168 patients who underwent ultrasound examination. Ethical approval from the institutional review board was secured, and all participants provided written informed consent. The study’s inclusion criteria were patients who (1) were preparing for thyroid surgery, (2) were willing to undergo two-dimensional ultrasound (2D-US) and 3D-US examinations, and (3) had nodules adjacent to the thyroid capsule. The exclusion criteria were as follows: (1) complete surgical and pathological records were not obtained (*n* = 4); (2) non-PTC patients (28 benign, 2 medullary carcinomas, and 3 follicular thyroid carcinomas); and (3) lymph node resection was not performed (*n* = 5); finally, 126 patients were included in this study.

### Image assessment

**Fig. 1:**
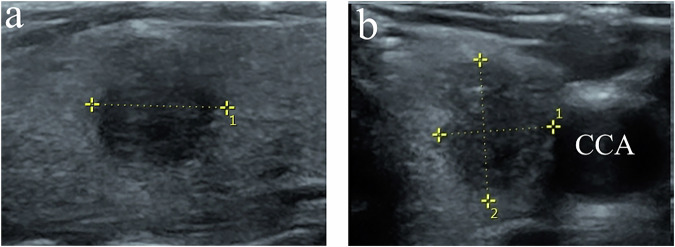
The longitudinal (**a**) and transverse (**b**) planes of 2D-US showed a 0.9 cm × 0.7 cm × 0.9 cm solid thyroid nodule in the left lobe of the thyroid. 2D-US, two-dimensional ultrasound; CCA, common carotid artery

**Fig. 2:**
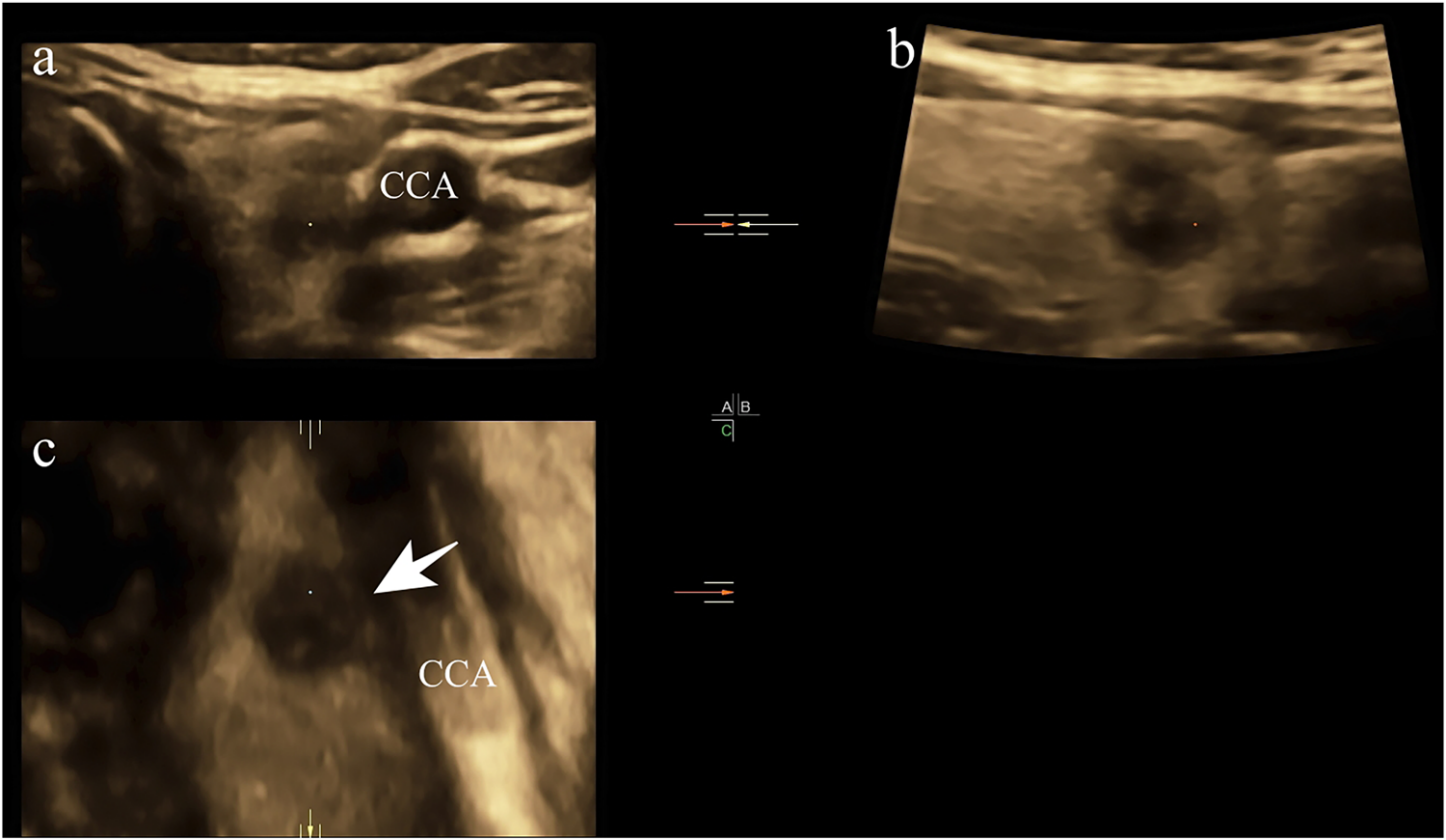
The multiplane of 3D-US showing the transverse (**a**), longitudinal (**b**), and coronal planes (**c**) of the above thyroid nodule in Fig. 1. In the **c** plane, the thyroid capsule near the CCA was interrupted (white arrow). 3D-US, three-dimensional ultrasound; CCA, common carotid artery

**Fig. 3:**
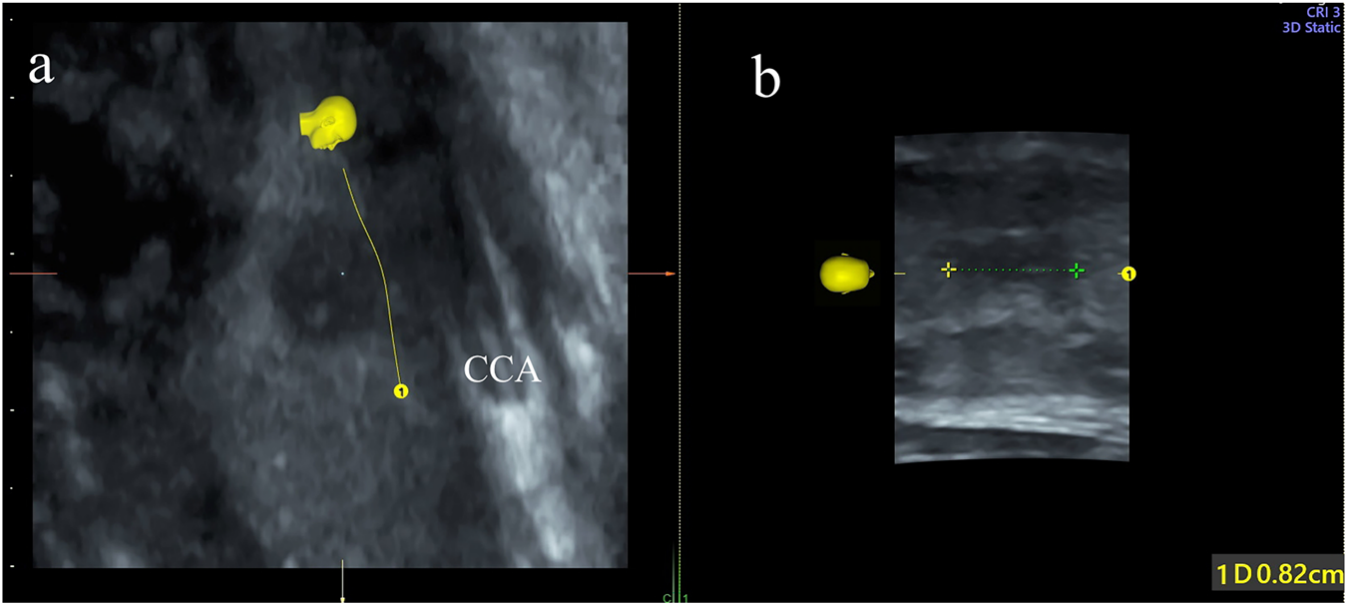
A polyline was drawn along the thyroid capsule near CCA in the coronal plane (**a**). The reconstructed 3D-OmniView plane showed a hypoechoic thyroid nodule extending into the hyperechoic thyroid capsule, and the LCD was 0.82 cm (**b**)

The 2D-US examination was performed with a 5–12 MHz broad-spectrum linear probe (iU22; Philips Healthcare). The 3D-US volume data were acquired with a 5–17-MHz broad-spectrum real-time 4D linear probe (GE Voluson E10; General Electric Medical Systems). While collecting 3D-US volume data, the probe was stabilized, the sweep angle was adjusted from 15° to 30° according to nodule size, and then the initial volume data were automatically acquired. The 3D-US scans were performed by one radiologist. The images were reviewed by two experienced radiologists with more than two years of experience in thyroid ultrasound. Discrepancies between the reviewers, such as the location of ETE and the plane showing the maximum length of capsular disruption, were resolved by consensus after joint re-evaluation of the images. Both reviewers were blinded to the patients’ information, including clinical history, previous radiological findings, and final diagnosis. We documented the clinical variables (such as sex and age), and ultrasonographic features (such as tumor size, multifocality, location, echogenicity, composition, shape, margin, echogenic foci, and thyroid capsule invasion), and performed malignancy risk stratification according to both the American College of Radiology Thyroid Imaging Reporting and Data System (ACR TI-RADS) [18] and the Chinese Thyroid Imaging Reporting and Data System (C-TIRADS) [19] (Fig. 1). In the analysis of the 3D-US data, suspicious ETE site was found in 3D-US (Fig. 2), and then a polyline was drawn along the interrupted thyroid capsule, a reconstructed warped plane of the capsule surface was built and defined as the 3D-OmniView plane, then the LCDs for all suspicious nodules were measured (Fig. 3). Additionally, we randomly selected 20 nodules suspicious for ETE. Two radiologists, each with over five years of experience in thyroid ultrasound, performed LCD detection and assessed the consistency of measurements using 3D-Omniview.

### Statistical analyses

Descriptive data were reported as the mean and standard deviation ($\bar{{{\bf{x}}}}$ ±  **S**) or median and interquartile range, as appropriate. Categorical data were described as numbers and percentages. Chi-square tests, *T*-tests, and Mann‒Whitney tests were used to evaluate the statistical significance of the associations between clinical or ultrasonographic features and LNM. The cutoff values of clinical and ultrasonographic features were obtained by the receiver operating characteristic (ROC) curve analysis. Pathological findings of LNM were criteria for diagnosing ETE. The statistically significant variables in the univariate analysis were subsequently included in the binary logistic regression analysis to construct a risk prediction model-nomogram. The ROC curve and calibration curve were used to evaluate the model’s discrimination and calibration, respectively. Intraclass correlation coefficient (ICC) was employed to evaluate the agreement of LCD measurements between the two radiologists. A *p* value less than 0.05 was considered to indicate statistical significance. Statistical analysis was performed using SPSS 26 software and R software.

## Results

### Clinical and ultrasound characteristics between LNM and no-LNM

Among 126 patients, there were 71 with LNM, 93 who underwent total thyroidectomy, 33 who had lobectomy, 98 who had central LND, and 28 who had both central and lateral LND. The average age of the LNM group (20–59 years) was lower than the non-LNM group (23–65 years) (*p* = 0.004). The proportion of males with LNM was higher than that of females (*p* = 0.009). Patients with larger malignant nodules, a larger sum of malignant nodules, larger LCD, echogenic foci, or thyroid capsule invasion were more likely to have LNM (*p* < 0.001, *p* < 0.001, *p* = 0.001, *p* = 0.026, *p* = 0.023). No significant differences were found in multifocality, location, echogenicity, composition, shape, and margin, with the highest ACR TI-RADS or C-TIRADS classifications of nodules between PTC patients with or without LNM. The ICC value was 0.952 (95% CI: 0.883–0.981), demonstrating excellent inter-evaluator agreement on LCD measurement (Table 1).

**Table 1:** Clinical and ultrasound characteristics between LNM and no-LNM

	No LNM (*n* = 55)	LNM (*n* = 71)	*p*
Age (mean ± SD, years)	45.98 ± 9.68	40.47 ± 9.99	0.004
Gender			0.009
Male	6 (10.9%)	22 (31.0%)	
Female	49 (89.1%)	49 (69.0%)	
Multifocality			0.425
Negative	42 (76.4%)	49 (69.0%)	
Positive	13 (23.6%)	22 (31.0%)	
Location of the largest suspicious nodule			0.456
Upper third	9 (16.4%)	17 (23.9%)	
Middle third	27 (49.1%)	38 (53.5%)	
Lower third	18 (32.7%)	15 (21.1%)	
Isthmus	1 (1.8%)	1 (1.4%)	
Echogenicity			0.355
Hyperechoic	1 (1.8%)	0 (0.0%)	
Isoechoic	0 (0.0%)	1 (1.4%)	
Hypoechoic	47 (85.5%)	65 (91.5%)	
Very hypoechoic	7 (12.7%)	5 (7.0%)	
Composition			0.411
Mixed cystic and solid	1 (1.8%)	3 (4.2%)	
Solid	54 (98.2%)	68 (95.8%)	
Shape			0.256
Wider-than-tall	21 (38.2%)	22 (31.0%)	
Taller-than-wide	34 (61.8%)	49 (69.0%)	
Margin			0.330
Smooth	4 (7.3%)	8 (11.3%)	
Lobulated or irregular	51 (92.7%)	63 (88.7%)	
Echogenic foci			0.026
None or large comet-tail artifacts	20 (36.4%)	12 (16.9%)	
Macrocalcifications	7 (12.7%)	7 (9.9%)	
Peripheral(rim) calcifications	0 (0.0%)	0 (0.0%)	
Punctate echogenic foci	28 (50.9%)	52 (73.2%)	
Thyroid capsule invasion			0.023
Negative	34 (61.8%)	30 (42.3%)	
Positive	21 (38.2%)	41 (57.7%)	
MDLM [M (Q1, Q3), cm]	0.90 (0.70, 1.10)	1.20 (0.80, 1.70)	< 0.001
SMDM [M (Q1, Q3), cm]	0.90 (0.70, 1.40)	1.45 (1.00, 2.08)	< 0.001
LCD [M (Q1, Q3), cm]	0.30 (0.00, 0.68)	0.61 (0.33, 1.01)	0.001
ACR TI-RADS			0.698
TR4	4 (7.3%)	3 (4.2%)	
TR5	51 (92.7%)	68 (57.1%)	
C-TIRADS			0.440
TIRADS 4a	0 (0.0%)	1 (1.4%)	
TIRADS 4b	8 (14.5%)	5 (7.0%)	
TIRADS 4c	45 (81.8%)	63 (88.7%)	
TIRADS 5	2 (3.6%)	2 (2.8%)	
Extent of surgery			0.001
Lobectomy	23 (41.8%)	10 (14.1%)	
Total thyroidectomy	32 (58.2%)	61 (85.9%)	
Lymphadenectomy			0.009
Central lymph nodes	49 (89.1%)	49 (69.0%)	
Central and lateral lymph nodes	6 (10.9%)	22 (31.0%)	

*LNM* lymph node metastasis, *MDLM* maximum diameters of the largest malignant nodule, *SMDM* sum of the maximum diameters of all malignant nodules, *LCD* length of capsule disruption, *ACR TI-RADS* American College of Radiology Thyroid Imaging Reporting and Data System, *C-TIRADS* Chinese Thyroid Imaging Reporting and Data System

### Development of a nomogram for predicting LNM in PTC patients

Age, gender, echogenic foci, thyroid capsule invasion, SMDM, maximum diameter of the largest malignant nodule (MDLM), and LCD were significant in univariate analysis (Table 1). Separately, ROC curve analysis of these continuous variables for predicting LNM, the cutoff value for LCD was calculated to be 0.42 cm, the cutoff values for MDLM and SMDM were 0.95 cm and 1.05 cm, respectively, and the cutoff value of age was 44.5-years-old (Table 2). Finally, aged more than 45-years-old, male, ETE, MDLM ≥ 1.0 cm, and LCD ≥ 0.42 cm were included in the multivariate analysis regression for assessment as risk factors of LNM (Table 3). SMDM, echogenic foci, and thyroid capsule invasion were removed from forward stepwise regression analysis. The logistic prediction model was developed as follows: logit(*p*) = −0.993 − 1.083 × age + 1.624 × gender + 1.276 × MDLM + 1.320 × LCD (0 for age < 45years, 1for age ≥ 45 years; 0 for female, 1 for male; 0 for MDLM < 1.0 cm, 1 for MDLM ≥ 1.0 cm; 0 for LCD < 0.42 cm, 1 for LCD ≥ 0.42 cm).

**Table 2:** The Cutoff value, AUC and OR of significant continuous variables for predicting LNM

	Cutoff value	AUC	OR (95%CI)	*p*
LCD	0.42 cm	0.673 (0.578–0.767)	4.097 (1.922–8.733)	< 0.001
MDLM	0.95 cm	0.706 (0.616–0.795)	4.220 (1.994–8.930)	< 0.001
SMDM	1.05 cm	0.697 (0.606–0.788)	3.803 (1.788–8.091)	0.001
Age	44.5 years	0.357 (0.261–0.454)	0.409 (0.199–0.842)	0.015

*LNM* lymph node metastasis, *MDLM* maximum diameters of the largest malignant nodule, *SMDM* sum of the maximum diameters of all malignant nodules, *LCD* length of capsule disruption, *OR* odds ratio

**Table 3:** Multivariate logistic regression for LNM of PTC

	β (SE)	*p* values	OR	95% CI of OR
Age ≥ 45 years	−1.083 (0.435)	0.013	0.339	0.144–0.795
Male	1.624 (0.565)	0.004	5.075	1.676–15.366
MDLM ≥ 1 cm	1.276 (0.449)	0.004	3.581	1.486–8.629
LCD ≥ 0.42 cm	1.320 (0.463)	0.004	3.742	1.511–9.266
Constant	−0.993 (0.437)			

*LNM* lymph node metastasis, *MDLM* maximum diameters of the largest malignant nodule, *LCD* length of capsule disruption, *OR* odds ratio

**Fig. 4:**
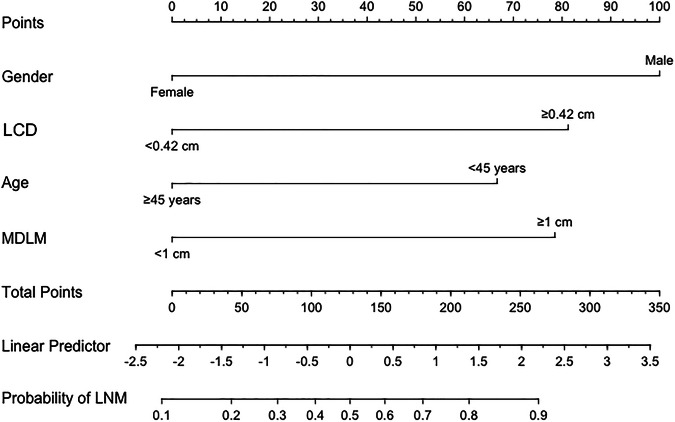
Nomogram for predicting LNM in PTC patients

A nomogram was created from important factors linked to LNM. The nomogram contained four risk factors (gender, age, MDLM, and LCD) to estimate the metastasis risk of LNM for PTC before surgery. Gender yields the largest contribution to the prediction model, while LCD provides the next largest contribution. We assigned a score to every level within variables based on the point scale. Subsequently, we determined the risk of CLNM in each subject by summing up all total scores and identifying it on the total point scale (Fig. 4).

### Evaluation of the clinical prediction model

**Fig. 5:**
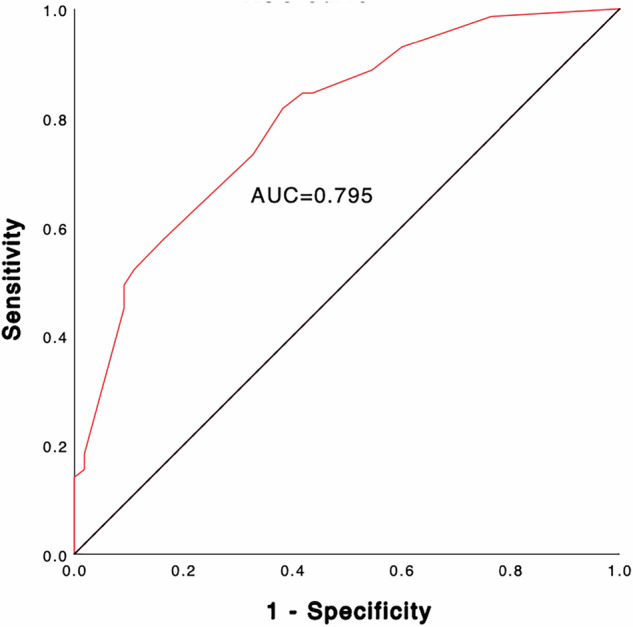
ROC curve analysis to predict LNM in PTC patients. ROC, receiver operating characteristic; AUC, area under the ROC curve

**Fig. 6:**
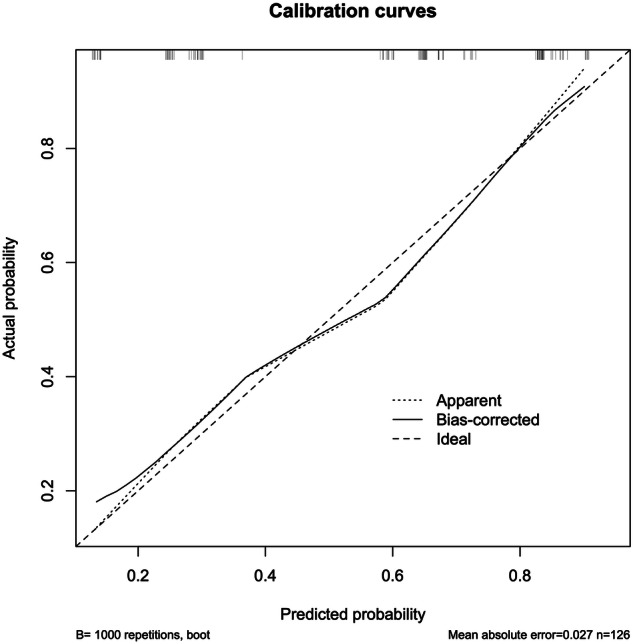
Calibration curve of the nomogram

The nomogram yielded the area under the ROC curve (AUC) 0.795 (95% CI: 0.718–0.873) with sensitivity, specificity, and accuracy as 58.2%, 84.5% and 73.0%, which indicated that the prediction model was good and had good clinical predictive ability (Fig. 5). The calibration curve was drawn by the bootstrap 1000 resampling method. The calibration curve mostly showed that there was no agreement between the predicted and actual results of the nomogram model, indicating that the calibration degree of the model was good (Fig. 6).

## Discussion

This study revealed a correlation between LCD and LNM in PTC patients. Patients with LCD ≥ 0.42 cm exhibit a 4.097-fold increased risk of LNM. Additionally, age, gender, MDLM, and LCD were identified as independent risk factors for LNM. The nomogram constructed in this study integrates these factors to aid in the preoperative prediction of LNM risk, offering more accurate personalized treatment recommendations. For example, the nomogram assigns a 40-year-old woman with a nodule MDLM ≥ 1 cm and LCD ≥ 0.42 cm a probability of LNM greater than 90%. In such cases, we recommend that sonographers remain vigilant and meticulously scan for metastatic lymph nodes, while surgeons should perform intraoperative gross lymph nodes exploration and consider prophylactic LND to obviate reoperation for recurrence. Conversely, patients at low risk of central lymph-node metastasis should not undergo prophylactic dissection, as this may cause unnecessary neck trauma and potential surgical complications.

The diagnosis of LNM in thyroid cancer is challenging due to atypical imaging features of early metastatic nodes, limitations in ultrasound examination of certain anatomical areas (e.g., retropharyngeal, retrosternal, and mediastinal regions), and significant variability in diagnostic accuracy based on operator experience [20]. These factors contribute to the limited diagnostic efficiency of preoperative US and CT, as evidenced by a meta-analysis of 5656 thyroid cancer patients showing pooled sensitivities of 0.593 for US and 0.664 for CT in diagnosing cervical LNM, with even lower sensitivities for central cervical LNM (0.284 for US and 0.400 for CT) [10]. In clinical practice, this leads to either over-treatment (e.g., unnecessary prophylactic LND causing complications such as permanent hypoparathyroidism) or under-treatment (e.g., insufficient LND requiring secondary or multiple surgeries), both of which negatively impact patient outcomes. Guidelines emphasize addressing this balance: the 2015 American Thyroid Association (ATA) guidelines recommend against routine central LND in low-risk differentiated thyroid cancer (DTC) to reduce permanent hypoparathyroidism by 50% without compromising oncologic outcomes [9], while the 2019 European Society for Medical Oncology (ESMO) guidelines mandate therapeutic lateral LND for clinically suspected N1b disease to lower reoperation rates by 70% and improve 10-year recurrence-free survival (RFS) by 15% [21]. Given these challenges, exploring risk factors for cervical LNM adequately and developing accurate clinical prediction models is essential. Our study addresses this by re-evaluating the controversial role of ETE as an LNM risk factor from the novel perspective of LCD, integrating clinical and sonographic features to develop a clinically practical prediction model.

According to the eighth edition of the American Joint Committee on Cancer (AJCC) guidelines, ETE is categorized as minimal and gross. Minimal ETE refers to the tumor only invading into and around the surrounding peri-thyroid soft tissues. Gross ETE refers to the tumor invading into and around the trachea, larynx, surrounding musculature, and vasculature [22]. Although the impact of minimal ETE on PTC clinical outcomes remains debated [23], some studies have identified minimal ETE as an independent predictor of persistent/recurrent disease and it is associated with LNM and a lower disease-free survival rate [24, 25]. In some studies, the postoperative pathologic result of ETE is used to predict the risk of LNM [26], while it is more helpful to predict LNM preoperatively. However, the diagnostic criteria for ETE on ultrasound vary across studies [13–17]. Our study aimed to use LCD as a quantitative measure to assess the risk of ETE in predicting LNM. We found that when LCD exceeds 0.42 cm, the risk of LNM increases 4.097-fold (OR = 4.097). In Mao’s meta-analysis, 9369 PTC patients with 37.17% LNM were analyzed, the pooled OR for capsular invasion in predicting LNM is 3.48, and the pooled OR for ETE in predicting LNM is 2.03 [12]. In our study, patients with LCD ≥ 0.42 cm had a 30.3% (23/76) rate of no LNM, while 36.0% (18/50) of patients with LCD < 0.42 cm had LNM. For thyroid capsule invasion, 38.2% (21/62) of positive cases had no LNM, and 42.3% (30/64) of negative cases had LNM. Compared to thyroid capsule invasion, LCD reduced the false-positive rate by 8.2% and the false-negative rate by 5.7%. This may suggest that LCD is a more accurate predictor of LNM. However, 36.0% of patients with LCD < 0.42 cm still had LNM, indicating potential underestimation. Using LCD alone may lead to unnecessary neck dissections or missed high-risk cases. Combining multiple indicators could improve prediction accuracy and clinical practice utility.

3D-US revealed a coronal plane that could not be visualized by 2D-US. The Omniview mode in 3D-US is an imaging technology that enables manual drawing of a line, curve, polyline, or trace from any direction or angle and presents the curved surface plane [27]. Ammar’s study has employed this technology to assess uterine wall defects [28]. Our previous research also demonstrated that the Omniview mode of 3D-US outperforms 2D-US in evaluating ETE in PTC and follicular thyroid carcinoma [29]. In this study, 3D-US technology, especially the Omniview mode, provides clearer visualization and more precise measurements, which are essential for preoperative assessment of ETE and prediction of LNM.

Compared with prior studies on cervical LNM in PTC, our results highlight the innovative value of 3D-US application and the use of LCD as a predictive factor. Generally, an AUC of 0.7–0.8 indicates good predictive performance of a model, while an AUC of 0.8–0.9 signifies excellent performance. Our study model included four parameters (sex, age, LCD, and MDLM), achieving an AUC of 0.795. In contrast, Lu’s study [30] incorporated four parameters (sex, age, thyroid capsule invasion, and lymph node microcalcification), yielding an AUC of 0.800. Li’s study [31] included six parameters (sex, age, largest diameter, capsular invasion, highest ACR score, and total ACR score), obtaining an AUC of 0.838 for the modeling group and 0.697 for the external test group. Sun’s study [6] integrated seven parameters (sex, age, US-reported cervical lymph node status, multifocality, tumor size, microcalcification, and ETE), achieving an AUC of 0.839. Actually, in clinical practice, when there are suspicious malignant features of lymph nodes, we generally perform preoperative biopsy or intraoperative LND. Therefore, an LNM prediction model is more meaningful for cases without positive imaging features of LNM. So our study did not include ultrasonographic features related to LNM.

The limitations of this study include constraints on sample size, study population, and equipment. With only 126 patients involved, although the results are statistically significant, further validation in larger, multicenter studies is needed. Moreover, in our study, only 51 patients had central LNM, 1 patient had lateral LNM, and 19 patients had both central and lateral LNM, so we didn’t separately evaluate LCD’s predictive value for lateral and central LNM. Additionally, LNM diagnosis relied on postoperative findings, possibly missing metastatic lymph nodes not removed during surgery and underestimating factors contributing to cervical LNM risk. Future research directions include expanding the sample size, conducting multicenter studies, and performing external validation of the nomogram. Long-term follow-up is also recommended to evaluate the model’s potential for assessing postoperative recurrence risk. What’s more, although the OmniView mode of 3D-US provides clearer visualization and more precise measurements, which are essential for preoperative assessment of ETE and prediction of LNM, it is not widely used across clinical departments. Currently, it is primarily utilized for US examinations of obstetrics and gynecology, rarely applied in thyroid imaging. Its application in superficial organs requires a superficial small organ 3D ultrasound probe, which may restrict the generalizability of our findings. Although 2D-US cannot display the coronal plane, meaning that longitudinal LCD measurements on the vascular and tracheal sides are limited and the maximal ETE may not be fully visualized, it can still display ETE on the anterior and posterior capsules, as well as the vascular and tracheal sides in the transverse view. In such cases, using curved measurements for LCD can still yield relatively accurate results. Future research could explore the potential of conventional 2D-US in measuring LCD as a predictor of LNM.

## Conclusion

In conclusion, this study underscores the clinical value of 3D-US in quantifying ETE and predicting LNM. By translating LCD into a reproducible metric, our nomogram bridges the gap between histopathological ETE classifications and preoperative ultrasound imaging, offering a pragmatic tool for risk-adapted surgery. Prospective validation in diverse populations is essential to confirm its utility in guiding therapeutic decisions.

## Abbreviations

2D-US: Two-dimensional ultrasound
3D-US: Three-dimensional ultrasound
ACR TI-RADS: American College of Radiology Thyroid Imaging Reporting and Data System
AUC: Area under the ROC curve
CCA: Common carotid artery
C-TIRADS: Chinese thyroid imaging reporting and data system
ETE: Extrathyroidal extension
LCD: Length of capsule disruption
LND: Lymph node dissection
LNM: Lymph node metastasis
MDLM: Maximum diameter of the largest malignant nodule
OR: Odds ratio
PTC: Papillary thyroid carcinoma
ROC: Receiver operating characteristic
SMDM: Sum of the maximum diameters of all malignant nodules
US: Ultrasound
